# Proteome profiling of human placenta reveals developmental stage-dependent alterations in protein signature

**DOI:** 10.1186/s12014-021-09324-y

**Published:** 2021-08-09

**Authors:** Sara Khorami Sarvestani, Sorour Shojaeian, Negar Vanaki, Behrouz Ghresi-Fard, Mehdi Amini, Kambiz Gilany, Hale Soltanghoraee, Soheila Arefi, Mahmood Jeddi-Tehrani, Amir-Hassan Zarnani

**Affiliations:** 1 Reproductive Biotechnology Research Center, Avicenna Research Institute, ACECR, Tehran, Iran; 2 Monoclonal Antibody Research Center, Avicenna Research Institute, ACECR, Chamran High Way, Evin, Tehran, Iran; 3 Department of Biochemistry, School of Medical Sciences , Alborz University of Medical Sciences, Karaj, Iran; 4 Department of Immunology, School of Public Health, Tehran University of Medical Sciences, Enghelab Ave, Tehran, Iran; 5 Department of Immunology, School of Medicine, Shiraz University of Medical Sciences, Shiraz, Iran; 6 Immunology Research Center, Iran University of Medical Sciences, Tehran, Iran

**Keywords:** Placenta, Proteomics, First-trimester, Full-term, 2D LC–MS/MS

## Abstract

**Introduction:**

Placenta is a complex organ that plays a significant role in the maintenance of pregnancy health. It is a dynamic organ that undergoes dramatic changes in growth and development at different stages of gestation. In the first-trimester, the conceptus develops in a low oxygen environment that favors organogenesis in the embryo and cell proliferation and angiogenesis in the placenta; later in pregnancy, higher oxygen concentration is required to support the rapid growth of the fetus. This oxygen transition, which appears unique to the human placenta, must be finely tuned through successive rounds of protein signature alterations. This study compares placental proteome in normal first-trimester (FT) and term human placentas (TP).

**Methods:**

Normal human first-trimester and term placental samples were collected and differentially expressed proteins were identified using two-dimensional liquid chromatography-tandem mass spectrometry.

**Results:**

Despite the overall similarities, 120 proteins were differently expressed in first and term placentas. Out of these, 72 were up-regulated and 48 were down-regulated in the first when compared with the full term placentas. Twenty out of 120 differently expressed proteins were sequenced, among them seven showed increased (GRP78, PDIA3, ENOA, ECH1, PRDX4, ERP29, ECHM), eleven decreased (TRFE, ALBU, K2C1, ACTG, CSH2, PRDX2, FABP5, HBG1, FABP4, K2C8, K1C9) expression in first-trimester compared to the full-term placentas and two proteins exclusively expressed in first-trimester placentas (MESD, MYDGF).

**Conclusion:**

According to Reactome and PANTHER softwares, these proteins were mostly involved in response to chemical stimulus and stress, regulation of biological quality, programmed cell death, hemostatic and catabolic processes, protein folding, cellular oxidant detoxification, coagulation and retina homeostasis. Elucidation of alteration in protein signature during placental development would provide researchers with a better understanding of the critical biological processes of placentogenesis and delineate proteins involved in regulation of placental function during development.

**Supplementary Information:**

The online version contains supplementary material available at 10.1186/s12014-021-09324-y.

## Introduction

Rearrangement of placenta in molecular, histological and functional aspects throughout pregnancy is fundamental for appropriate fetal development and maternal health. Out of about 12,000 genes expressed in human placenta, the majority of genes change their expression patterns during pregnancy indicating a preplanned rearrangement scenario needed to adapt to the changing demands of the fetus [1–3]. Of note, most changes in gene expression occur during the first-trimester placenta (FT) versus term placenta (TP). Comparative analysis of gene expression in the villous tissues of first and second trimester versus TP showed that early gestational-age placenta is characterized by higher expression of genes involved in cell proliferation and apoptosis. In this regard, increased Wnt pathways activity in 1st and 2nd trimester is consistent with proliferative activity and invasiveness of trophoblasts. This increased expression of genes and pathways involved in promoting cell proliferation is accompanied with increased expression of cyclin dependent kinase (CDK) inhibitors which is likely an important mechanism to control proliferative activity of trophoblasts [4]. Besides cell proliferation, cell differentiation is another hallmark of placental development and is accompanied by profound changes in molecular signature. Differentiation of proliferative cytotrophoblasts (CTBs) into extravillous trophoblasts (EVTs) during placental development involves a series of well-defined molecular alterations including down regulation of E-cadherin and α6β4 integrin, and upregulation of VE-cadherin, α5β1, αV family members, platelet endothelial cell adhesion molecule-1 (PECAM-1), and vascular cell adhesion molecule 1 (VCAM-1) as well as matrix metalloproteinase-9 [5]. CTB differentiation also entails the modulation of Notch family members and modulation of several growth factors and receptors including vascular endothelial growth factor (VEGF). In first-trimester, the majority of placental growth and development occurs under low oxygen tension which is an important modulator of invasive EVT proliferation, differentiation, invasion and angiogenesis [5–7]. Oxygen regulates cellular proteome and function through the hypoxia-inducible factor (HIF) [8, 9] which is responsible for the hypoxic induction of hundreds of genes related to angiogenesis trophoblast differentiation and invasion by binding to hypoxia response elements (HREs) in their promoters or enhancers [7, 8, 10].

To gain a better insight into the molecular mechanisms involved in development of placenta during pregnancy, identification of human placental proteome and proteins differentially expressed at different stages of placenta development is necessary. While genome is relatively static, proteome is highly variable and could potentially give rise to identification of higher degrees of alteration [11]. Several reports have been published so far to compare the placental proteome in normal placentation and pregnancy-related disorders. Mine et al. reported the human placental proteome map using whole full TP in 2007 using two-dimensional electrophoresis followed by identification by MALDI-TOF [12]. Consequently, Mushahary et al. reported a group of new proteins in human term placental proteome [13].

There is a myriad of reports in the literature on placental proteome. Some of these reports have investigated proteome profile of placental cell lines [14–16], while the others reported data on placental proteins of non-human species [17–19]. Reports on proteome of human placental tissue can be generally classified into two main categories; (1) whole or sub-proteome profiling of either first or term placenta [20–23], (2) comparative proteome analysis of term placenta in normal versus a diseased condition such as pre-eclampsia, diabetes, fetal growth restriction, and Down’s syndrome [12, 15, 24–30]. However, data on comparative proteome analysis of normal first and third-trimester placentas is elusive. We previously reported total placental proteome differences between first and third-trimester human placentas using 2D-PAGE-MALDI-TOF. In that report, normal FT placentas were selected from late FT pregnant women referring to the legal abortion committee, due to mother indication for abortion such as heart disease [31]. Here we extend our previous observation by proteome profiling of totally normal first and third-trimester human placentas using two-dimensional electrophoresis followed by identification by LC-ESI MS/MS. FT placentas were collected from women with normal unintended pregnancy during voluntary termination of pregnancy. Term placentas were collected from women with cesarean delivery who delivered normal male babies. All participants had a normal course of pregnancy. The results presented here could provide researchers with deeper insights into molecular and cellular processes during placental development.

## Materials and methods

### Placental samples

In this study, four normal human FT whole placentas and four TP were collected. All procedures were carried out in accordance with the ethical committee of Avicenna Research Institute (ARI) (ethical approval No: 1397.007) and with the revised version of Helsinki Declaration in 2013. Written informed consents were obtained from all subjects before clinical sampling. All participants had a history of at least one previous successful healthy pregnancy and delivery and were selected from Caucasians living in Tehran/Iran after matching their ages (30 ± 2 and 33 ± 2 years for first trimester and term placenta donors, respectively). The normal FT placentas were taken from healthy pregnant women during voluntary termination of unintended pregnancy. TPs were obtained at cesarean section. All aspects of pregnancy health including systolic blood pressure, body mass index (BMI), mother and fetus weight, blood glucose level and obesity were continuously monitored during the course of pregnancy. All pregnant women had no history of abortion; chronic or acute illness; used no medications before caesarian section or induced abortion. To minimize the potential impact of sex on proteome profile, all TPs belonged to male fetuses, however, the gender of aborted fetuses was unclear (gestational age < 12w). The mean gestational age of FT placentas were 10 ± 2 weeks and those of TPs were 38 ± 1 week. Placental samples analyzed in the FT and TP were just 4 per group. Difficulties in finding placenta donors which met inclusion criteria for further processing and analysis led to limitation in sample size per group.

All placenta samples were analyzed by a pathologist and confirmed to be normal. The placentas were quickly put in cold phosphate-buffered saline (PBS) after collection, kept on ice and immediately transferred to the laboratory. From each placenta, four samples with 1 cm thickness from four directions (including both maternal and fetal sides) and one sample from the central part were cut using a sterile scalpel and pooled. The weight of each wet punch was about one gram. The pooled samples were then washed several times in cold PBS to eliminate the contaminating blood, aliquoted and stored in liquid nitrogen until protein extraction. The same procedure was applied for all placentas (four FT and four TP).

### Protein extraction and quantification

Four placentas were collected for each first (FT) and third trimesters (TP). Five pieces from both maternal and fetal sides were punched from each placenta, mixed and frozen. Four frozen first trimester- and four frozen term placenta samples were separately mixed to have FT and TP pools, respectively. The pooled samples were pulverized by cryogenic grinding with liquid nitrogen using a chilled mortar and pestle (Additional file 1: Fig. S1). Pulverized sample (0.1gr) was homogenized in 1 mL lysis buffer containing 8 M urea, 2% w/v CHAPS, 2% dithiothreitol (DTT) in 5 mM Tris–HCl pH 7.6 and incubated on ice for 15 min with gentle vortexing. Homogenate was centrifuged at 15,000*g* for 1 h at 4 °C. Supernatant was collected and its protein concentration was determined by 2-D Quant Kit (GE Healthcare, USA). Aliquoted samples were then stored at − 20° C until being analyzed by 2D-PAGE.

### Two-dimensional PAGE

Initially, the first dimension (isoelectric focusing, IEF) of 2D-PAGE was carried out using 17 cm linear immobilized pH gradient (IPG) strip with pH 3–10 (Bio-Rad) and processed. Based on the protein distribution pattern, the differences in proteome of FT and TP were mostly localized between pH range of 5 to 8. To this end, all subsequent experiments were done using 17 cm linear IPG strip with pH 5–8 (Bio-Rad). FT and TP protein extracts were run and stained simultaneously in a twin gel electrophoresis system (Bio-Rad) to minimize the variations. In-gel rehydration of IPG strips was done by using 200 and 500 μg protein extract for Colloidal Coomassie Stain (CCS) and silver nitrate staining, respectively. Protein sample was mixed with rehydration buffer (8 M urea, 2% CHAPS, 2% DTT, 2% IPG buffer and 0.001% bromophenol blue) to a final volume of 300 μl. After rehydration (~ 17 h at room temperature), isoelectric focusing (IEF) was performed on the strips at 20° C to reach a total of 50,000 Vh (PROTEAN IEF Cell, Bio-Rad). Subsequently, the strips were equilibrated in equilibration buffer (6 M urea, 50 mM Tris–HCl pH 8.8, 30% v/v glycerol, 2% SDS, and 0.001% bromophenol blue) containing 60 mM dithiothreitol (DTT) for 15 min at 37° C followed by another 15 min incubation in equilibration buffer containing 135 mM iodoacetamide (IAA) at room temperature. Next, a twin gel electrophoresis system (PROTEAN II xi Cell, Bio-Rad) was used for the second dimension. The strips were placed on 8–15% gradient SDS-PAGE gels, sealed using 1% agarose, and run first at a constant voltage of 30 V for 15 min followed by 45 V constant voltage for the next ~ 12 h until the front line reached the bottom of the gels.

### 2D gels staining and spot selection

First and third-trimester placenta protein 2D gels were stained with CCS or silver nitrate. 2D image scanner and image master 2D platinum software, v 6 (Pharmacia, Uppsala, Sweden) were used for scanning gels and spot analysis, respectively. The same parameters were used for all gels to avoid variation in analyses. A single master gel image containing all spots was prepared in each group as the master gel. After determining the percentage of intensity (% intensity) for each spot, the mean intensities of the same spots on different gels were compared by student T-test using EXCEL software (Microsoft, version 2010) program and p values < 0.05 were considered statistically significant. Among the 120 DEPs, 20 proteins with higher score including 18 proteins with dual expression patterns in both FTs and TPS and 2 proteins with unique expressions in FTs were selected for LC–MS/MS analysis. These spots were carefully punched out of CCS-stained gels followed by in-gel digestion as described by Shevchenko et al. [32]. Gel pieces were dehydrated in 50% Acetonitrile and rehydrated in 50 mM Tris pH8.0 + 10 mM DTT. Pieces were heated for 15 min at 65 °C. Bands were then reduced by adding 15 mM iodoacetamide (IAA) and incubated 30 min in the dark at room temperature. Remaining IAA was quenched by the addition of 10 mM DTT. Pieces were dehydrated once more with 50% Acetonitrile and rehydrated in a Trypsin/LysC solution. Digestion was carried out overnight at 37 °C. Peptides were purified by reversed phase extraction and analyzed by LC–MS.

### LC–MS/MS parameters

For LC–MS/MS, acquisition was performed with an ABSciex TripleTOF 5600 (ABSciex, Foster City, CA, USA) coupled to an electrospray interface with a 25 μm iD capillary and connected to an Eksigent μUHPLC (Eksigent, Redwood City, CA, USA). Analyst TF 1.7 software was adapted to control the apparatuses and data processing and acquisition. For the IDA mode, the source voltage was set to 5.5 kV and maintained at 325 °C. Curtain gas pressure was set at 27 psi, gas one at 27 psi, and gas two at ten psi. The separation was done on a reversed-phase HALOC18-ES column 0.3 μm, i.d., 2.7 μm particles, 150 mm long (Advance Materials Technology, Wilmington, DE) and maintained at 60 °C. Samples were injected by loop overfilling into a 5μL loop. For the 60 min [33] LC gradient, the mobile phase consisted of solvent A (0.2% v/v formic acid and 3% DMSO v/v in water) and solvent B (0.2% v/v formic acid and 3% DMSO in EtOH) at a flow rate of 3 μl/ min [32, 33].

### Bioinformatics analysis

All data from LC–MS/MS runs were analyzed simultaneously with the Protein Pilot software to identify candidate proteins. To further investigate the biological significance of twenty differentially expressed proteins, we carried out GO and pathway enrichment analyses employing different online databases and software. For GO, Shiny GO v0.61 (http://bioinformatics.sdstate.edu/go) and PANTHER v15 (http://www.pantherdb.org) were used and pathway enrichment analysis was done using Reactome database (http://reactome.org). R software (version 3.6.1, https://www.r-project.org) and packages GOplot and ggplot2 were applied for visualization of GO terms and enriched pathways, respectively.

## Results

### Placenta collection and determination of protein concentration

In this study, normal human placentas from FT and TP were collected and sampled. In each FT and TP group, samples from four placentas were pooled, tissue lysates were prepared and the protein concentrations were determined. On average, from 0.1 g of FT and TP tissues 4.42 ± 1.65 and 3.64 ± 0.59 mg proteins were extracted, respectively. The protein concentrations of placental lysate aliquots were measured at different time points after preparation and were always shown to be consistent.

### Quantification of proteins in placenta lysate

The protein concentration of prepared placenta lysates was determined by 2-D Quant Kit. On average, from 0.1 g of FT and TP tissues 4.42 ± 1.65 and 3.64 ± 0.59 mg protein were extracted, respectively. The protein concentration of placental lysate aliquots was measured at different time points after preparation and always was shown to be consistent.

### Comparative proteome analysis between FTs and TPs

**Fig. 1:**
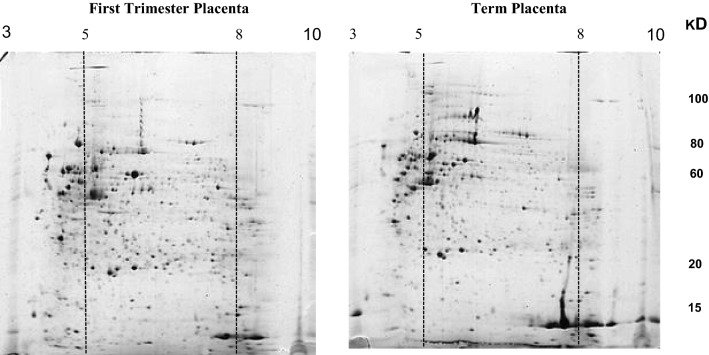
2D gel electrophoresis of human placenta. Protein lysate from normal human FT and TP was separated in first dimension on IPG strip (17 cm, pI range 3–10) and in the second dimension by SDS-PAGE (8–15%gradient gel). Gels were stained with colloidal Coomassie stain. Differential protein spots were mostly localized in pI range of 5–8. FT: First trimester placenta; TP: Term placenta

**Fig. 2:**
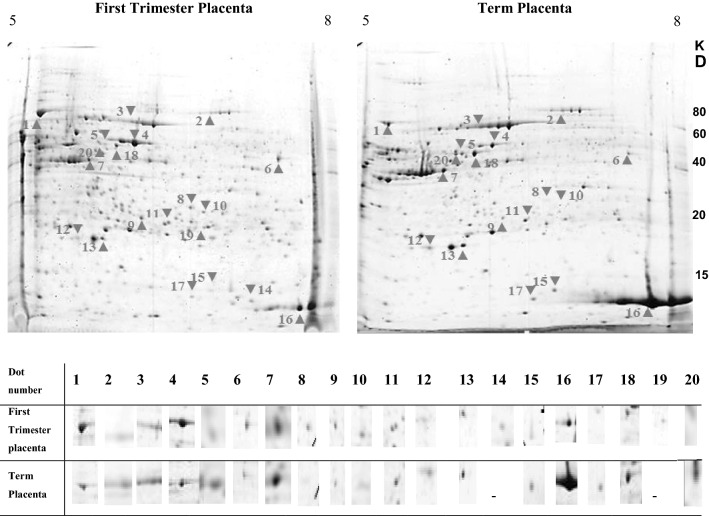
2D gel electrophoresis of differentially expressed proteins in FT and TP. Proteins were separated in first dimension on IPG strip (17 cm, pI range 5–8) and in the second dimension by SDS-PAGE (8–15%gradient gel). Gels were stained with colloidal Coomassie stain and differentially expressed proteins (DEP) were identified using image master 2D platinum software. Twenty DEP (arrowhead) were selected and subjected to mass spectrometry (LC–MS/MS). The picture depicts representative image of four independent experiments. FT: First trimester placenta; TP: Term placenta

At first, placental proteins were electrophoretically separated in pH range of 3–10. We observed that most placental protein spots were concentrated in areas pH range of 5–8, although there were a not negligible number of spots in the 4–5 range. Indeed, this was the pH range of 5–8 where differentially expressed proteins in the FT and TP, were mostly localized (Fig. 1). In this regard, all subsequent analyses were performed in pH range of 5–8. Accordingly, a total of 1262 and 1095 spots were expressed in FT and TPs, respectively. Despite the overall similarity, comparison of the percentage of dot intensities between FT and TP gels revealed a total of 513 matched dots. Among the total matched dots, 120 spots were differentially expressed. Out of the 120 spots, 72 had increased expression of more than twofold and 48 showed decreased expression of less than 0.5 fold in FT compared with TP. These spots were carefully inspected by three independent observers and finally 20 spots, differentially expressed in FT and TP were selected for LC–MS/MS data analysis with high accuracy (External calibration: 1–2 ppm RMS) (Fig. 2). The identity of differentially expressed proteins (DEP) and peptide coverage for each protein are summarized in Table 1.

**Table 1:** Differentially expressed protein spots in the FT compared to the TP, identified by LC–MS/MS

Spot^a^	Protein name	Mr^b^	pI^b^	Unused^c^	%Cov (95)^d^	Accession No	Peptides (95%)^e^
1	78 kDa glucose-regulated protein OS = Homo sapiens GN = HSPA5 PE = 1 SV = 2	72.4/ 78	5.07/ 5.2	74.43	69.1	sp|P11021|GRP78 HUMAN	131
2	Serotransferrin OS = Homo sapiens GN = TF PE = 1 SV = 3	79.3/ 83	6.08/ 6.7	87.78	61.8	sp|P02787|TRFE HUMAN	56
3	Serum albumin OS = Homo sapiens GN = ALB PE = 1 SV = 2	67/ 75	5.7/ 6.1	88.09	73.4	sp|P02768|ALBU HUMAN	112
4	Protein disulfide-isomerase A3 OS = Homo sapiens GN = PDIA3 PE = 1 SV = 4	58/ 60	5.8/ 6.2	91.24	79.6	sp|P30101|PDIA3 HUMAN	231
5	Keratin, type II cytoskeletal 1 OS = Homo sapiens GN = KRT8 PE = 1 SV = 7	51.3/ 50	5.5/ 5.9	47.16	69.2	sp|P05787|K2C8 HUMAN	79
6	Alpha-enolase OS = Homo sapiens GN = ENO1 PE = 1 SV = 2	47.3/ 50	6.98/ 7.3	50	65.9	sp|P06733|ENOA HUMAN	57
7	Actin, cytoplasmic 2 OS = Homo sapiens GN = ACTG1 PE = 1 SV = 1	42.1/ 40	5.3/ 5.7	30.21	52.8	sp|P63261|ACTG _HUMAN	21
8	Delta(3,5)-Delta(2,4)-dienoyl-CoA isomerase, mitochondrial OS = Homo sapiens GN = ECH1 PE = 1 SV = 2	34.4/ 30	6.61/ 6.5	48.18	71.3	sp|Q13011|ECH1 HUMAN	92
9	Peroxiredoxin-4 OS = Homo sapiens GN = PRDX4 PE = 1 SV = 1	24.9/ 27	5.73/ 6.3	32.51	67.2	sp|Q13162|PRDX4 _HUMAN	102
10	Endoplasmic reticulum resident protein 29 OS = Homo sapiens GN = ERP29 PE = 1 SV = 4	28.5/ 25	6.7/ 6.5	31.12	61.3	sp|P30040|ERP29 HUMAN	35
11	Enoyl-CoA hydratase, mitochondrial OS = Homo sapiens GN = ECHS1 PE = 1 SV = 4	26.1/ 25	5.9/ 6.4	35.79	77.6	sp|P30084|ECHM HUMAN	65
12	Chorionic Somatomammotropin Hormone 2 OS = Homo sapiens GN = CSH2 PE = 1 SV = 1	18.8/ 25	5.34/ 5.6	23.98	62.7	sp|P0DML3|CSH2 HUMAN	56
13	Peroxiredoxin-2 OS = Homo sapiens GN = PRDX2 PE = 1 SV = 5	22.04/ 20	5.65/ 5.9	45.98	75.8	sp|P32119|PRDX2 HUMAN	69
14	Myeloid-derived growth factor OS = Homo sapiens GN = MYDGF PE = 1 SV = 1	18.7/ 17	6.31/ 7.1	22.63	56.7	sp|Q969H8|MYDGF HUMAN	32
15	Fatty acid-binding protein, epidermal OS = Homo sapiens GN = FABP5 PE = 1 SV = 3	15.16/ 16	6.6/ 6.9	15.88	60.7	sp|Q01469|FABP5 HUMAN	19
16	Hemoglobin subunit gamma-1 OS = Homo sapiens GN = HBG1 PE = 1 SV = 2	16.1/ 15	6.7/ 7.5	39.62	95.2	sp|P69891|HBG1 HUMAN	120
17	Fatty acid-binding protein,adipocyte OS = Homo sapiens GN = FABP4 PE = 1 SV = 3	14.71/ 16	6.59/ 6.9	26.15	66.7	sp|P15090|FABP4 HUMAN	35
18	Keratin, type II cytoskeletal 8 OS = Homo sapiens GN = KRT8 PE = 1 SV = 7	51.3/ 50	5.5/ 6	71.34	60.5	sp|P05787|K2C8 HUMAN	117
19	LDLR chaperone MESD OS = Homo sapiens GN = MESDC2 PE = 1 SV = 2	26.07/ 19	6.1/ 6.6	24.35	57.3	sp|Q14696|MESD HUMAN	39
20	Keratin, type I cytoskeletal 9 OS = Homo sapiens GN = KRT9 PE = 1 SV = 3	62.06/ 51	5.04/ 5.8	20.21	21.7	sp|P35527|K1C9 HUMAN	11

LC–MS/MS: Liquid chromatography-tandem mass spectrometry; Mr: the average mass of the protein; pI: isoelectronic point; FT: First trimester placenta; TP: Term placenta

^a^Spot numbers are the same as the spot labels in Fig. 2

^b^Theoretical/ Experimental Mr (KD); ^b^Theoretical/ Experimental pI

^C^Unused is the score computed by the software according to the number of good peptides (the higher the score, the higher the confidence that this protein was identified)

^d^ %Cov is the Coverage percentage

^e^Peptides(95%) is the number of the identified peptides with a confidence higher than 95%

As shown in Table 2, seven of the twenty DEP were up-regulated (GRP78, PDIA3, ENOA, ECH1, PRDX4, ERP29 and ECHM), while eleven proteins (TRFE, ALBU, ACTG, CSH2, PRDX2, FABP5, HBG1, FABP4, K2C8, K1C9 and K2C1) showed decreased expression in normal FTs compared to the normal TPs. Besides, two unique proteins were exclusively expressed in the FTs (MESD and MYDGF).

**Table 2:** Comparison of the mean percentage intensity of the differentially expressed spots between first trimester (First) and third trimester (Third) human placentas and their main biological process

No	Uniprot ID	Protein name	First	Third	Change^a^	Fold change^b^	Biological process	P value
1	P11021	GRP78	1.6276	0.6599	▲	2.466	Protein folding	1.515E-05
2	P02787	TRFE	0.1619	0.3618	▼	0.447	Hemostatic process	6.275E-04
3	P02768	ALBU	0.3066	0.7733	▼	0.396	Hemostatic process	7.095E-03
4	P30101	PDIA3	1.240	0.7803	▲	1.589	Protein folding	2.545E-03
5	P04264	K2C1	0.0988	0.3107	▼	0.317	Blood coagulation	9.41E-05
6	P06733	ENOA	0.4726	0.2174	▲	2.173	Cellular catabolic process	4.259E-04
7	P63261	ACTG	0.372	0.7053	▼	0.527	Blood coagulation	5.944E-03
8	Q13011	ECH1	0.169	0.0976	▲	1.731	Metabolic pathways	2.595E-03
9	Q13162	PRDX4	0.2158	0.0788	▲	2.738	Response to stress	6.958E-04
10	P30040	ERP29	0.1616	0.042	▲	3.847	Protein folding	7.616E-05
11	P30084	ECHM	0.1258	0.104	▲	1.210	Metabolic pathways	8.5655E-01
12	P0DML3	CSH2	0.207	0.376	▼	0.550	Metabolic pathways	1.192E-02
13	P32119	PRDX2	0.276	0.551	▼	0.500	Cellular response to external stimuli	5.214E-04
14	Q969H8	MYDGF	0.223	0.000	▲	####	Cellular response to external stimuli	7.942E-05
15	Q01469	FABP5	0.128	0.346	▼	0.369	Metabolic pathways	3.527E-02
16	P69891	HBG1	2.030	9.27	▼	0.218	Blood coagulation	2.182E-03
17	P15090	FABP4	0.040	0.32	▼	0.129	Metabolic pathways	1.275E-03
18	P05787	K2C8	0.273	0.760	▼	0.359	Programing cell death	8.455E-05
19	Q14696	MESD	0.120	0.000	▲	####	Protein folding	1.395E-02
20	P35527	K1C9	0.135	0.473	▼	0.285	Programing cell death	1.841E-05

^a^Up-regulation or down-regulation of the spots in the normal FT compared to TP indicated by ▲ and ▼, respectively

^b^Fold change in the normal FT compared to the TPs calculated based on exact P values by Mann–Whitney T test

### Bioinformatics analysis of differentially expressed proteins

**Fig. 3:**
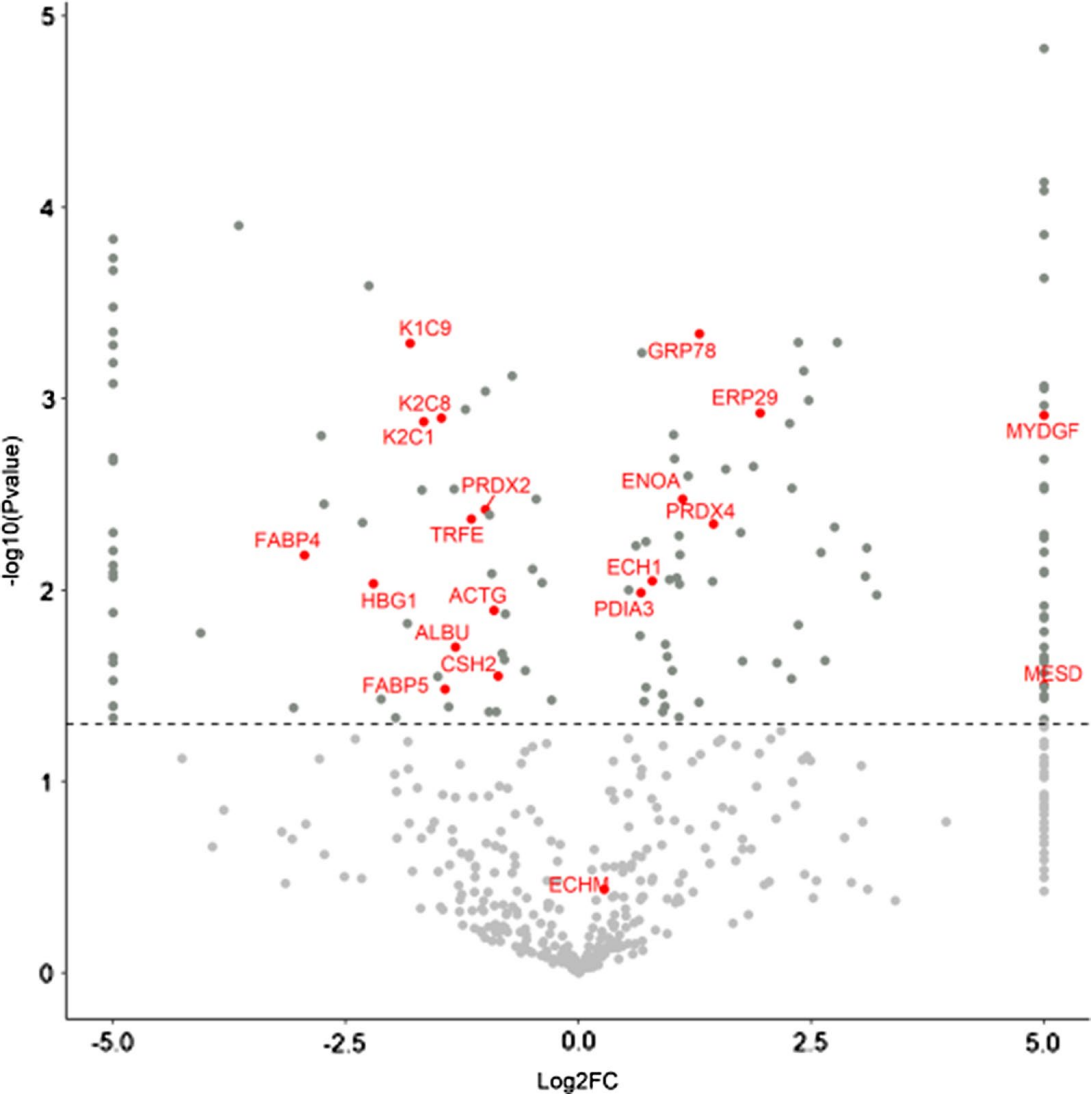
Volcano plot of quantitative placenta proteomic analysis of FT and TP. 513 matched protein dots in first and third- trimester placentas are shown in a Volcano plot. Statistical analysis was performed by student t-test, and statistical significance was considered when p < 0.05. Red dots represent identified proteins exhibiting significant fold changes (FC) in the normal FT compared to TP. Despite no significant fold change for ECHM, this spot was carefully selected in visual inspection of the stained gels. MYDGF and MESD were only expressed in first-trimester placentas and located at infinitive region of the plot. FT: First trimester placenta; TP: Term placenta

**Fig. 4:**
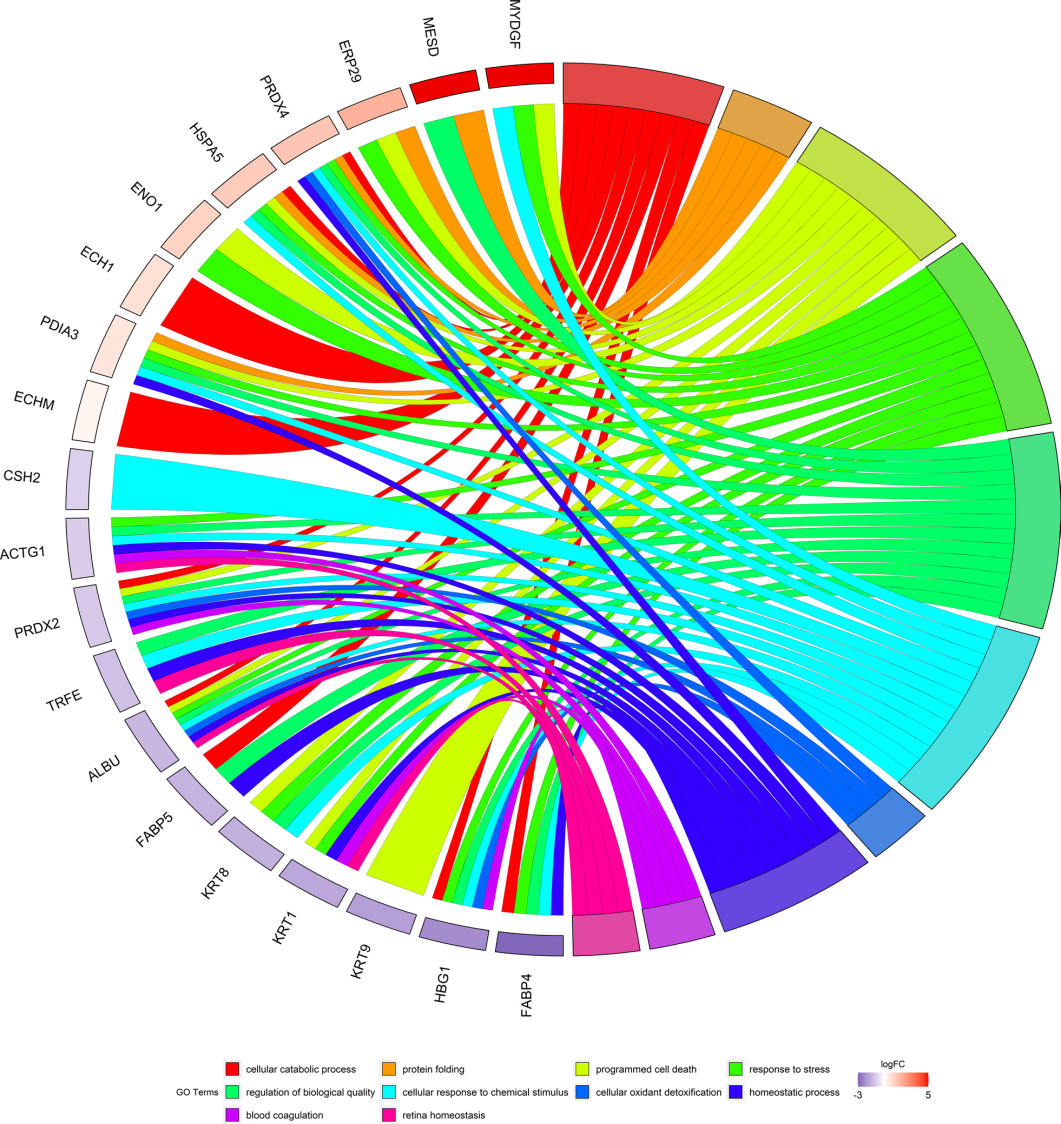

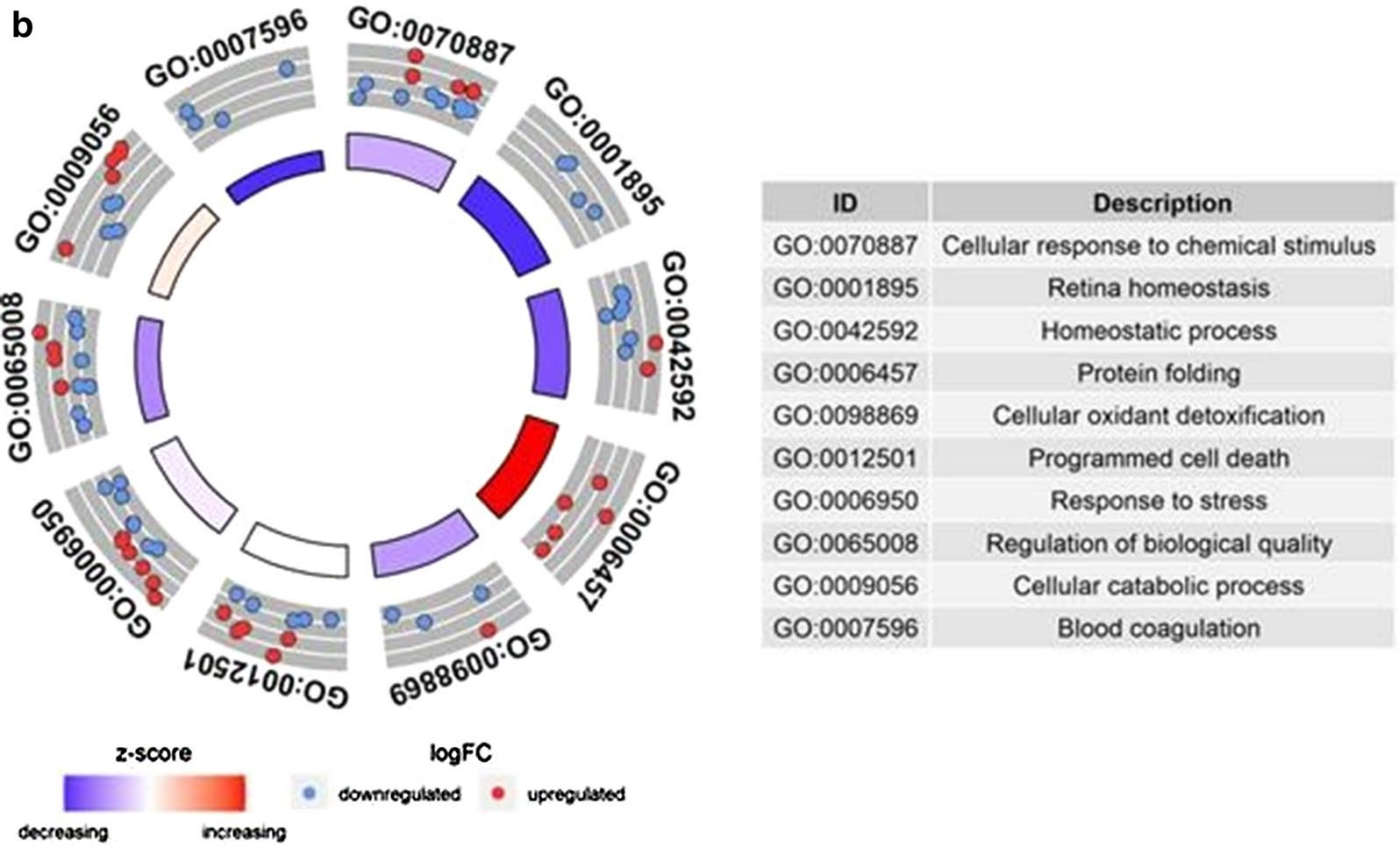
Biological procecesses of differentially expressed proteins in FT and TP: **A** GOChord plot of top 10 ranked overrepresented GO terms belonging to the biological process in 20 detected placenta proteins. The placenta proteins are linked to their assigned terms via colored ribbons. Placenta proteins were ordered according to the observed log-fold change of first trimester placenta compared to term one (logFC), which is displayed in descending intensity of red squares displayed next to the selected genes. Circle drawing represented detected proteins in the mainly involved biological processes. **B** The circle plot of the differently expressed proteins and z-scores of GO BP (bilogical process) terms. The outer circle shows a scatter plot of the assigned proteins. Red circles display up-regulation and blue ones down-regulation. The inner circle shows the z-score of each GO BP term. The width of each bar corresponds to the number of proteins within GO BP term and the color corresponds to the z-score

To further characterize the expression pattern of 120 DEPs, we visualized these proteins in Volcano plot (Fig. 3). Among them, 62 proteins were expressed both in FT and TPs. While, 35 and 22 protein spots were exclusively expressed in FTs and TPs, respectively. Moreover, one dot, Enoyl-CoA hydratase mitochondrial (ECHM), which was not significantly overexpressed in FT, was included in DEP list based on consensus of three independent observers. The identified 20 proteins were then subjected to GO using Pantherdb online software and functional pathway enrichment analysis. Based on this, proteins were involved mostly in protein processing in endoplasmic reticulum, signaling and metabolic pathways (Table 2) and engaged in ten biological functions including cellular response to chemical stimulus, response to stress, regulation of biological quality, programmed cell death, hemostatic process, cellular hemostatic process, cellular catabolic process, protein folding, retina hemostasis, cellular oxidant detoxification and blood coagulation. Moreover, the data analysis revealed that all detected proteins which were engaged in protein folding were over expressed in FT compared to TP, while those involved in blood coagulation and retina hemostasis were mostly down regulated in the FTs compared with the TPs (Fig. 4).

## Discussion

Adequate placental function is instrumental not only for developing embryo but also for its health after birth [34, 35]. In parallel with embryo development, the placenta undergoes considerable remodeling at different gestational stages. Proliferation of trophoblastic cells, for example, decreases from the third-trimester's onset until term, while the proliferation of endothelial cells increases [31, 36]. These consecutive changes at both molecular and cellular levels ensure constructive adaptations needed to fulfill the embryo's requirements at different pregnancy periods. Alterations in the placental proteome might accompany these changes. For instance, an environment with low O_2_ tension that prevails in the first trimester leads to an alteration in metabolic machinery accompanied by predominance of glycolysis and protection from damage by free radicals [37]. This cellular stress is the main trigger for secretion of myeloid-derived growth factor (MYDGF) protein to act as a paracrine/autocrine survival factor, a notion that justifies upregulation of this protein in FT [38]. It is the first report of differential expression of MYDGF protein in FT and TP. Cellular stress and inflammation is also associated with 78 kDa glucose-regulated protein (GRP78) expression with anti-inflammatory activity. GRP78 suppresses LPS-induced production of cytokines by promoting Toll-like Recepto (TLR4) internalization, during which CD14 is a crucial receptor for GRP78 [39]. Higher expression of GRP78 in FT correlates with its function to control inflammatory and stress conditions [40]. Several studies have also confirmed the upregulation of GRP78 in FT or placental function [13, 31, 41]. Peroxiredoxins (PRDXs) are a family of antioxidant proteins with six members, among which PRDX2 and PRDX4 are expressed by cytotrophoblast cells and play an essential antioxidant activity by detoxifying peroxides and as a sensor of hydrogen peroxide-mediated signaling events [28, 42–45]. Increased expression of PRDX4 in first trimester placentas is in line with its antioxidant activity. On the other hand, increased expression of PRDX2 in TP may be considered as a stress response to the inflammatory condition associated with the increased cortisol levels during labor. Expression of PRDX in the placenta has already been reported, but no comparison has been made between FT and TP so far [12, 13, 27, 46].

Metabolic adaptations are a crucial part of pregnancy, as they provide the mother with sufficient energy stores to meet pregnancy demands [47]. In first and second trimester placentas, the anabolic pathways prevail, while progression toward term is associated with a net catabolic phase with a breakdown of fat deposits to provide substrates for the growing fetus [48]. Here, proteins with crucial role in catabolism, fatty acid-binding protein, adipocyte (FABP4) and fatty acid-binding protein, epidermal (FABP5) were up regulated in TP, a finding which had already been reported in mouse placenta [49]. FABPs are required for placental preferential transport of maternal plasma long-chain polyunsaturated fatty acids during the last trimester to develop the brain and retina of the fetus [50]. Interestingly, pregnant women with high FABP4 levels in first trimester were more likely to develop preeclampsia [51, 52]. We also found differential expression of other key enzymes active in fatty acid metabolism in FT and TP. ECHM and delta (3,5)-delta (2,4)-dienoyl-CoA isomerase, mitochondrial (ECH1) up-regulated in FT compared to TP. These enzymes are essential molecules in fatty acids beta-oxidation pathway. In this regard, their upregulation in FT may be considered as a counter regulatory mechanism for increased anabolic events leading to accumulation of placental fatty acids during early gestational periods. Defects in beta-oxidation of fatty acids may result in pregnancy-related disorders like PE [12, 27]. We observed higher expression of chorionic somatomammotropin hormone 2 (CSH2) in TP. CSH2 is a placental polypeptide hormone which is secreted by syncytiotrophoblasts during pregnancy, and its structure and function are similar to the human growth hormone. It stimulates lactation, fetal growth and metabolism and regulates the metabolic state of the mother during pregnancy to facilitate the energy supply of the fetus. It can be detected from the sixth week of gestation, increases steadily in the first- and second-trimesters, and peaks at a constant level in the third-trimester consistent with its function to support fetal growth and metabolism [53].

Oxidative stress and inflammation are among potential causes of improper protein folding and aggregation. These events frequently occur during placental development, especially at early stages, and need to be corrected by compensatory molecular machinery. We found upregulation of five proteins with profound impact on protein folding in FT, LDLR chaperone MESD (MESD), GRP78 (HSPA5), protein disulfide-isomerase A3 (PDIA3), PRDX4 and Endoplasmic reticulum resident protein 29 (ERP29) which is in line with earlier reports. GRP78 is a heat shock protein which plays a crucial role in protein folding and quality control in the endoplasmic reticulum lumen. It is characterized as a p53 partner in trophoblastic cells and regulates trophoblastic invasion, an active process in the FT [31, 41]. For the first time we reported that MESD was expressed solely in FTs. MESD is a chaperone for the Wnt co-receptors: low-density lipoprotein receptor-related protein (LRP) 5 and 6 (LRP5/6). MESD is essential for differentiation of the epiblast, functions as a general LRP chaperone and its absence results in misfolding of multiple LRP receptors [54]. PDIA3 and ERP29 are involved in the ER stress signaling pathway which is known as the unfolded protein response [55, 56], which mostly occur during placental development. Expression of these proteins in term placenta has been reported earlier [13, 57].

During a healthy pregnancy the hemostatic balance changes in different trimesters. Uteroplacental circulation is not fully established until the end of the first-trimester [58]. From first-trimester to term placenta, hemostatic balance changes in favor of hypercoagulability, thus decreasing bleeding complications in connection with delivery. Our data on increased expression of proteins involved in blood coagulation in TP is in line with this notion. Iron metabolism is highly active in placenta. Iron is actively transferred from mother to the fetus, especially at later stages of fetus development. TRFE is responsible for iron transport in human term placenta [12, 13, 59]. However its expression is increased in placental abnormalities to fulfill an increased need for the TF function to meet the fetal iron needs [59, 60]. This data supports our finding of preferential expression of TRFE in TP.

Alpha-enolase (ENOA), also called non-neuronal enolase, belongs to a family of cytoplasmic and glycolytic enzymes and is involved in various processes such as growth control, hypoxia tolerance, and in the metabolism of carbohydrates [61]. Also ENOA, as a cell membrane plasminogen receptor, modulates fibrinolytic system [62] suggesting its role in reducing invasion and migration of trophoblasts by inhibiting the action of the fibrinolytic system. As we reported previously, it has higher expression in the FTs of pregnancy [31] which could be attributed to its role in glycolysis during first trimester.

## Conclusion

Despite several reports available in the literature about human placental proteome in a specified period of pregnancy and those associating protein expression changes with pregnancy-related diseases, data on comparative proteomic profiling of normal human placenta in first and term placenta is still elusive. Here, we employed 2-DE followed by LC–MS/MS to look at global differences in protein expression pattern in normal first versus term human placenta to identify differentially expressed proteins in either period. The findings of the present study introduced a group of 20 differentially expressed proteins, some of which were reported here for the first time. These proteins were mostly involved in response to chemical stimulus and stress, programmed cell death, hemostatic and catabolic processes, protein folding, cellular oxidant detoxification, and coagulation. Elucidation of alteration in protein signature during placental development would provide researchers with a better understanding of the critical biological processes of placentogenesis and delineate proteins involved in regulation of placental function during development.

## Supplementary Information

**Additional file 1: Figure S1.** Flow diagram of the experimental design. Four placentas were collected for each first (FT) and third trimesters (TP). Five pieces from both maternal and fetal sides were punched from each placenta, mixed and frozen. Four frozen first trimester- and four frozen term placenta samples were separately mixed to have FT and TP pools, respectively. The pooled samples were pulverized by cryogenic grinding with liquid nitrogen using a chilled mortar and pestle. The sample powders (0.1gr) were homogenized in 1 mL lysis buffer and analyzed using 2D SDS-PAGE. The 2D SDS-PAGE analyses were repeated in four independent experiments in each group (FT and TP). The spots were compared and then 20 differentially expressed spots were carefully punched out of CCS-stained gels followed by LC–MS/MS analysis.

## Data Availability

The datasets used and/or analyzed during the current study are available from the corresponding author on reasonable request.

## Abbreviations

FT: First-trimester placenta
TP: Term placenta
CDK: Cyclin dependent kinase
CTB_S_: Cytotrophoblasts
EVT_S_: Extravillous trophoblasts
PECAM-1: Platelet endothelial cell adhesion molecule-1
VCAM-1: Vascular cell adhesion molecule 1
VEGF: Vascular endothelial growth factor
HIF: Hypoxia-inducible factor
HREs: Hypoxia response elements
BMI: Body mass index
PBS: Phosphate-buffered saline
CCS: Colloidal Coomassie Stain
IEF: Isoelectric focusing
DTT: Dithiothreitol
IAA: Iodoacetamide
GRP78: 78 kDa glucose-regulated protein
TRFE: Serotransferrin
ALBU: Serum albumin
PDIA3: Protein disulfide-isomerase A3
K2C8: Keratin, type II cytoskeletal 1
ENOA: Alpha-enolase
ACTG: Actin, cytoplasmic 2
ECH1: Delta (3, 5)-Delta (2, 4)-dienoyl-CoA isomerase, mitochondrial
PRDX4: Peroxiredoxin-4
ERP29: Endoplasmic reticulum resident protein 29
ECHM: Enoyl-CoA hydratase, mitochondrial
CSH2: Chorionic Somatomammotropin Hormone 2
PRDX2: Peroxiredoxin-2
MYDGF: Myeloid-derived growth factor
FABP5: Fatty acid-binding protein, epidermal
HBG1: Hemoglobin subunit gamma-1
FABP4: Fatty acid-binding protein, adipocyte
K2C8: Keratin, type II cytoskeletal 8
MESD: LDLR chaperone MESD
K1C9: Keratin, type I cytoskeletal 9
DEP: Differentially expressed proteins
