# Supplementary figures and images for: Proteome profiling of human placenta reveals developmental stage-dependent alterations in protein signature
Source: Clin Proteomics. 2021 Aug 9;18:18. doi: 10.1186/s12014-021-09324-y (PMC8351416; doi:10.1186/s12014-021-09324-y)

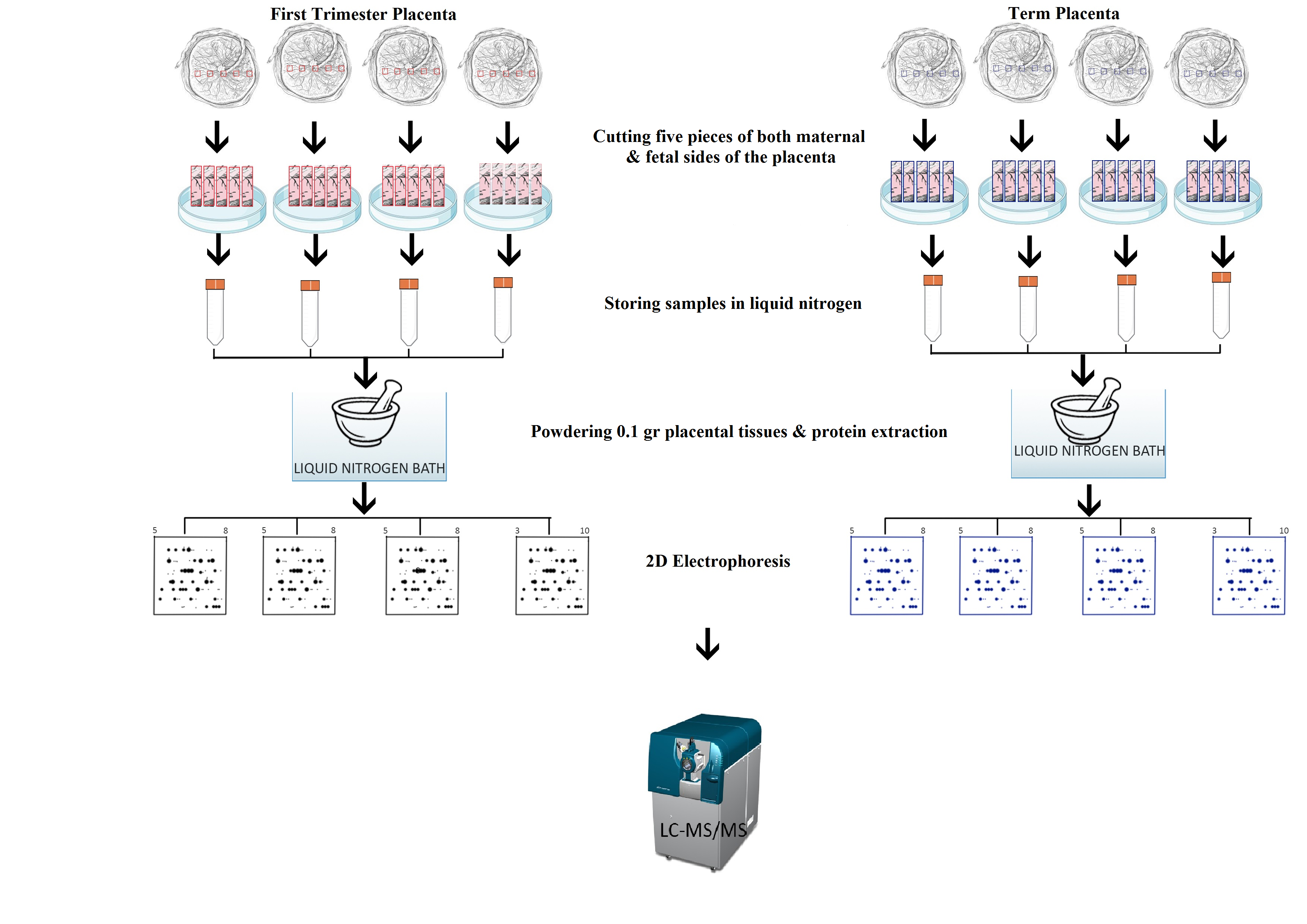

Supplement: Supplementary file 1 — Additional file 1: Figure S1. Flow diagram of the experimental design. Four placentas were collected for each first (FT) and third trimesters (TP). Five pieces from both maternal and fetal sides were punched from each placenta, mixed and frozen. Four frozen first trimester- and four frozen term placenta samples were separately mixed to have FT and TP pools, respectively. The pooled samples were pulverized by cryogenic grinding with liquid nitrogen using a chilled mortar and pestle. The sample powders (0.1gr) were homogenized in 1 mL lysis buffer and analyzed using 2D SDS-PAGE. The 2D SDS-PAGE analyses were repeated in four independent experiments in each group (FT and TP). The spots were compared and then 20 differentially expressed spots were carefully punched out of CCS-stained gels followed by LC–MS/MS analysis. [file 12014_2021_9324_MOESM1_ESM.tif]
